# Behavioural responses to potential dispersal cues in two economically important species of cereal-feeding eriophyid mites

**DOI:** 10.1038/s41598-017-04372-7

**Published:** 2017-06-20

**Authors:** Agnieszka Kiedrowicz, Lechosław Kuczyński, Mariusz Lewandowski, Heather Proctor, Anna Skoracka

**Affiliations:** 1 Population Ecology Lab, Faculty of Biology, Adam Mickiewicz University in Poznań, Umultowska 89, 61-614 Poznań, Poland; 2 Department of Applied Entomology, Faculty of Horticulture, Biotechnology and Landscape Architecture, Warsaw University of Life Sciences - SGGW, Nowoursynowska 159, Warsaw, 02-776 Poland; 3 Department of Biological Sciences, University of Alberta, Edmonton, Alberta T6G 2E9 Canada

## Abstract

Passively dispersing organisms should optimise the time and direction of dispersal by employing behaviours that increase their probability of being successfully transported by dispersal agents. We rigorously tested whether two agriculturally important passively-dispersing eriophyoid species, wheat curl mite (WCM) and cereal rust mite (CRM), display behaviours indicating their readiness to depart from current host plants in the presence of potential dispersal cues: wind, an insect vector and presence of a fresh plant. Contrary to our expectations, we found that both species decreased their general activity in the presence of wind. When exposed to wind, WCM (but not CRM) significantly increased behaviour that has previously been considered to facilitate dispersal (in this case, standing vertically). Our study provides the first sound test of the function of what have been interpreted as dispersal-related behaviours of eriophyid mites. The low proportion of WCM exhibiting dispersal behaviour suggests there may be predisposed dispersers and residents in the population. Moreover, we found that WCM was generally more active than CRM, which is likely a contributing factor to its high invasive potential.

## Introduction

Dispersal is a fundamental biological process that has consequences for population dynamics, population genetic structure, and geographical distribution of species^1–5^. At the individual level, dispersal can influence fitness by reducing inbreeding and competition among kin, by distributing offspring from the same parents over different conditions (‘bet hedging’), or by allowing individuals to escape unfavourable local conditions^6, 7^. However, this process also involves costs that can be incurred at every stage of dispersal, i.e., departure, transit, and settlement^2^. Costs of dispersal may differ in actively vs. passively dispersing organisms. An actively dispersing organism can control its own movement and can make decisions about timing, direction and speed in all three stages of dispersal. In contrast, in passively dispersing organisms, direction and speed of movement are largely outside of their control and depend instead on external forces such as gravity, wind or water currents, or on other organisms that act as vectors^6^. In particular, passive dispersers cannot control the transit and settlement stages of dispersal. However, they may partially control departure, e.g., by making the decision whether to take off and by engaging in behaviours that increase the possibility of being lifted by dispersal agents^8^. Existence of directed dispersal in passively dispersing organisms has recently been proposed for plants^9^. As another example, ‘pre-ballooning’ behaviours in spiders increase the probability of their being picked up by the wind, and include raising the abdomen, producing silk and jumping^10^. Additionally, spiders can choose the time of ballooning based on wind conditions^11^ and cloud cover^12^. Pre-dispersal postures have also been well documented in scale insect (Coccoidea)^13^, predatory phytoseiid mites^14–16^ and phytophagous tetranychid spider mites^17–19^, which additionally exhibit collective behaviours facilitating dispersal^20, 21^, and have been proposed to occur in phytophagous eriophyoid mites^22^.

**Figure 1:**
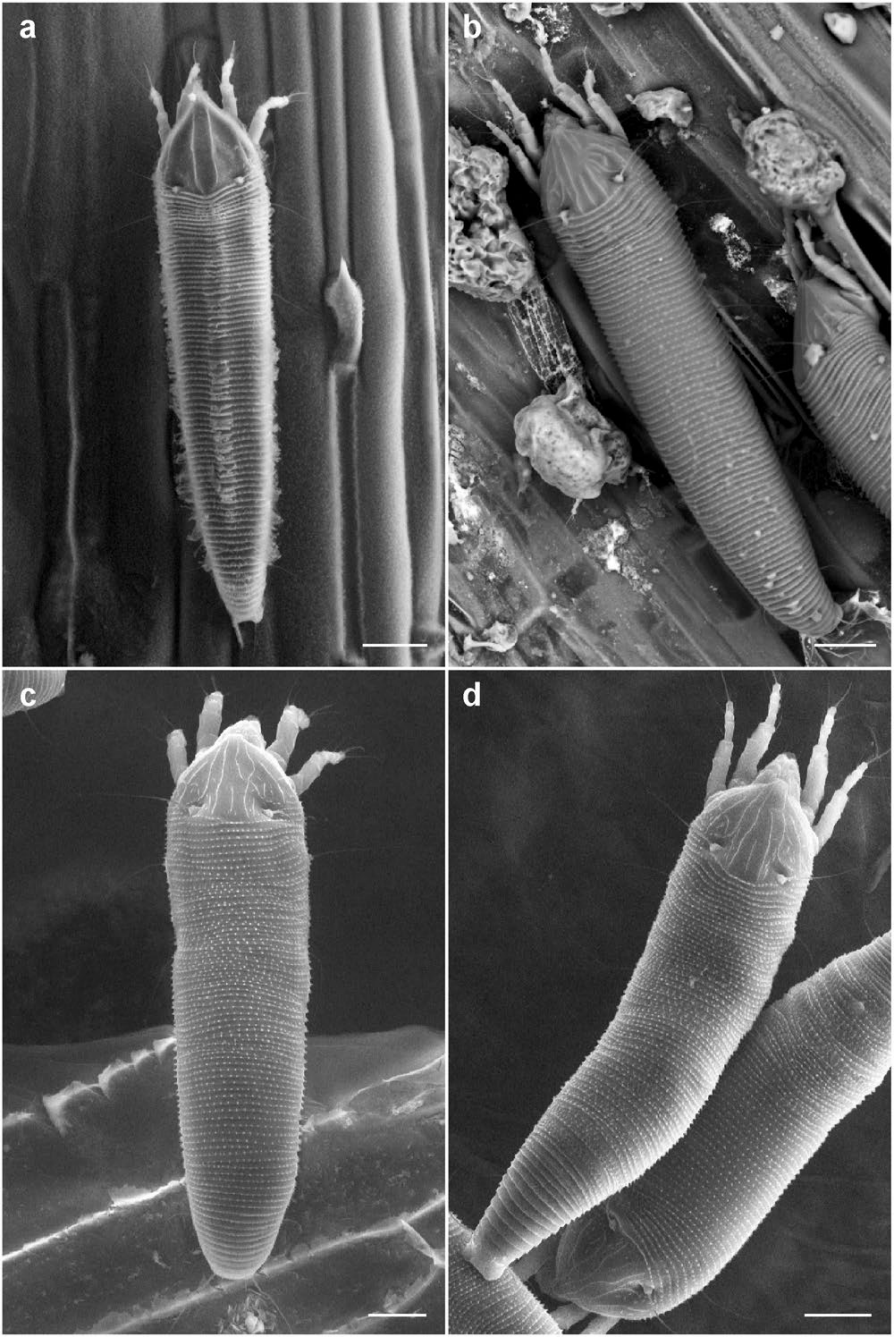
SEM images of eriophyid mites. Scale bars are 10 μm on each photo. (**a**) – Wheat curl mite (*Aceria tosichella* WCM MT-1 genotype) on wheat leaf; (**b**) – Cereal rust mite (CRM, *Abacarus hystrix*) on wheat leaf; (**c**) – standing-erect mite belonging to the WCM complex; (**d**) – chain- forming mites belonging to the WCM complex.

Eriophyoid mites (Eriophyidae, Phytoptidae and Diptilomiopidae) are obligatory plant parasites that are limited in ambulatory dispersal due to their extremely small size (most are less than 300 µm long), vermiform body shape, and possession of only two pairs of short anteriorly positioned legs (Fig. 1a and b). Passive aerial or phoretic dispersal has been suggested as the major way that eriophyoids spread^22, 23^. Behaviours that have been interpreted as facilitating dispersal in these mites include standing up on their anal lobes, arching their bodies and moving their legs rapidly, raising the hind part of the body while standing on their forelegs (all of which have been termed ‘take-off postures’), as well as forming groups of attached individuals (chains)^23–26^ (Fig. 1c and d). Additionally, these ‘take-off postures’ have been observed in eriophyoid mites when on an unsuitable host plant species and have been interpreted as indicating host-rejection and intent to disperse^27^.

The decision whether or not to disperse is a trade-off between risky movement and possible benefits from successful colonization^1^. The energetic investment in dispersal behaviours or the production of drifting structures can be costly^10, 28, 29^. Dispersal may expose individuals to predators (mortality costs)^30^. Finally, making an incorrect decision and taking off under unsuitable environmental conditions may increase the mortality during transit or settlement^31^. Direct mortality costs in aerially dispersing arthropods may increase with prolonged time spent in the air due to depletion of energy and water reserves^10^. Eriophyoid mites are especially at risk of dispersal-associated starvation as they are relatively host-specific and cannot survive for long if they land on an inappropriate plant species^23^.

Although it has been well documented that departure-specific behaviours affect the likelihood of aerial dispersal of spider mites^19, 32^, there has been no empirical evidence that similar behaviours in eriophyoid mites promote the probability of their becoming airborne or being picked up by vector organisms. Melo *et al*.^33^ found that aerial dispersal in the eriophyid coconut mite, *Aceria guerreronis* Keifer, is only occasionally preceded by a raised body stance at take-off. They concluded that raised body position may assist departure but it is not a prerequisite for take-off. It has also been argued that eriophyoid mites are simply blown off accidentally from their host plants and that they may have no specific behavioural adaptations to promote dispersal^33, 34^. Conversely, increased walking activity in the presence of a suitable host plant, due to chemical cues released by the plant, has been suggested as an important mode of eriophyoid dispersal^22, 23^. Additionally, this increased activity may increase the chance of the mite being picked up by the wind and potentially being blown to the nearby plant. Undoubtedly, the question whether eriophyoid mites have evolved any specific behaviours playing a role in their dispersal and colonization success remains unanswered.

Here we test whether specific departure behaviours occur in two very tiny (body length ca. 0.20–0.25 mm) cereal-feeding eriophyid mites by investigating their behavioural responses to the presence of three potential dispersal signals: wind, insects that could act as vectors, and fresh foliage from a suitable host plant. We used two economically important species with worldwide distributions: *Aceria tosichella* Keifer, wheat curl mite (WCM), and *Abacarus hystrix* (Nalepa) (*sensu lato*), cereal rust mite (CRM). Both belong to species complexes consisting of lineages that can be distinguished principally by differences in cytochrome c oxidase subunit I (COI) gene fragment^35, 36^. We tested the hypothesis that in the presence of dispersal cues mites display behaviours indicating their readiness to disperse, including increasing walking activity, initiating take-off postures and forming chains of individuals. Additionally we examined whether the particular behaviours shown by the mites are influenced by the type of dispersal cue or rather are invariant within a species, and whether responses to cues differ between the two species.

## Results

All mite behaviours (feeding, walking, standing erect and chain formation) were strongly and significantly influenced by species and treatment, but were not influenced by their interaction (Table 1).

**Table 1:** Analysis of deviance table testing influence of treatment, species and their interaction (dispersal cue x species) on mite behavioural responses.

Behavioural response	variable	Df	LR Chi square	p
Feeding	dispersal cue	3	42.7	<0.0001
	species	1	257.0	<0.0001
	dispersal cue × species	3	5.1	0.1674
Walking	dispersal cue	3	57.7	<0.0001
	species	1	258.9	<0.0001
	dispersal cue × species	3	7.1	0.0674
Standing erect	dispersal cue	3	31.1	<0.0001
	species	1	9.2	0.0024
	dispersal cue × species	3	5.1	0.1632
Chain formation	dispersal cue	3	17.1	0.0007
	species	1	14.3	0.0001
	dispersal cue × species	3	6.5	0.0912

Model terms, test statistics and associated *p*-values are given.

**Figure 2:**
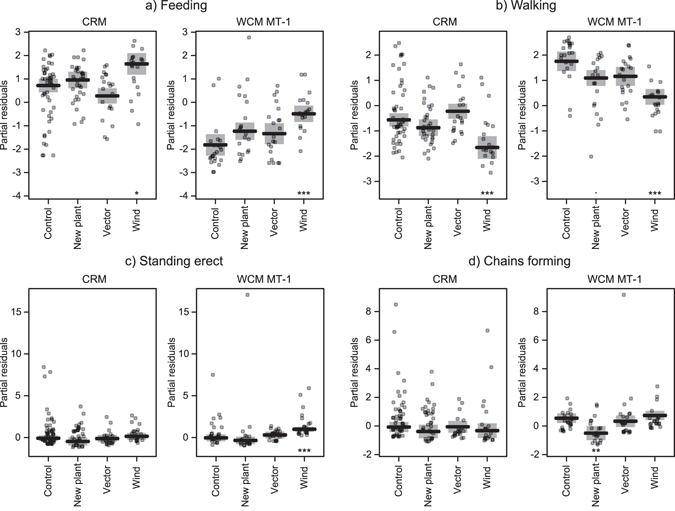
Results of Simultaneous Tests for General Linear Hypotheses. Effects of potential dispersal cues on the proportion of mites displaying particular behaviours (feeding, walking, standing erect, chain formation) when comparing to the control for the two species of eriophyid mites. Values on vertical axes are partial residuals. Significance codes: ***p < 0.001; **p < 0.01; *p < 0.05; ^·^p < 0.1. Exact p values can be found in Supplementary Table S2.

Wind significantly influenced the proportions of mites feeding and walking for both species, and the proportion standing erect for WCM (Fig. 2, see also Supplementary Tables S1 and S2). Higher probabilities of feeding in CRM (83.3%, 95% CI = 76.1–89.2%) and WCM (37.2%, 95% CI = 29.4–45.5%) but lower probabilities of walking in CRM (14.1%, 95% CI = 9.1–20.3%) and WCM (55.0%, 95% CI = 47.3–62.5%) were observed in the presence of wind when compared to other treatments. The probability of WCM standing erect in the presence of wind was low (4.0%, 95% CI = 3.1–5.2%), but nonetheless was significantly higher when compared to the proportion standing erect in other treatments (Fig. 2, see also Supplementary Table S2). The presence of fresh plant material significantly influenced WCM behaviour resulting in lower probability of mites forming chains (0.9%, 95% CI = 0.5–1.4%) compared to other treatments (Fig. 2, Supplementary Table S2). There was no significant behavioural response of mites to the presence of a potential insect vector (the thrips *Anaphothrips obscurus* Müller) (Fig. 2).

Overall, CRM had a higher proportion of individuals feeding (68.5%, 95% CI = 64.2–72.6%), but lower proportions walking (29.5%, 95% CI = 25.7–33.5%), standing erect (1.4%, 95% CI = 1.1–1.7%), and forming chains (1.2%, 95% CI = 0.9–1.5%) than did WCM (feeding: 23.3%, 95% CI = 19.6–27.3%; walking: 70.9%, 95% CI = 66.9–74.7%; standing erect: 2.1%, 95% CI = 1.7–2.6%; chain formation: 2.0%, 95% CI = 1.6–2.4%).

## Discussion

Passive dispersal by wind or by larger organisms is a common phenomenon in wingless, small-bodied organisms, which are not able to control the direction of their movement once airborne or on the body of a vector^37, 38^. However, timing of the launch phase of passive dispersal can potentially be actively controlled by the dispersing individuals^8^. In this study we analyzed the behaviours that have been long suggested to facilitate passive dispersal in eriophyoid mites, the smallest plant-feeding arthropods. The three potential dispersal cues tested in our study reflect three possible modes of eriophyid mite dispersal: (i) air dispersal: wind; (ii) active walking: fresh plant presence; (iii) phoresy: insect vector presence. We hypothesized that mites would change their behaviour by increasing walking activity, initiating take-off postures and forming chains of individuals when dispersal cues were present. However, contrary to our expectation, mites did not increase walking in the presence of any of the potential dispersal cues, and moreover they did not respond at all to the insect vector. Dispersal by biotic vectors has been suggested as the dispersal mode in eriophyid mites by several authors, as eriophyoid mites have sometimes been found attached to the bodies of larger arthropods^24, 39–42^. Moreover, Liu *et al*.^43^ found very strong evidence for obligate phoresy of an eriophyid mite, *Aceria pallida* Keifer, which uses the psyllid *Bactericera gobica* (Loginova) as a vector when moving to its winter hibernation sites on its woody perennial host. One of our test species, the wheat curl mite (WCM), has been found on thrips and aphids (reviewed in Michalska *et al*.)^22^. However, some authors have doubted that the phoresy plays a major role in the spreading of eriophyoid mites. Lindquist & Oldfield^34^ pointed out that eriophyoids do not show any clear morphological adaptations for attaching to hosts, and Galvão *et al*.^44^ did not find efficient dispersal of eriophyoids with insect vectors. Our results also indicate the lack of evident behavioural adaptations to phoretic dispersal. It is possible that attaching to insect vectors by the studied grass-feeding eriophyoid mites (reviewed in Michalska *et al*.^22^ as well as our own observations) is accidental rather than intentional. However, even if accidental, such attachment might occasionally result in dispersal of eriophyoids to new areas. It is also possible that phoresy may be common in eriophyoid mites inhabiting long-lived hosts (such as *A*. *pallida* mentioned above) and accidental or very rare in eriophyoids associated with short-lived hosts, such as grasses.

The mode of movement that has been suggested as the most efficient for eriophyoid mites is dispersal by wind currents^23, 34^, similar to plant-associated spider mites^45, 46^ and phytoseiid mites^15^. Indeed, our study suggests that wind was the main factor changing mite behaviour in both studied species. However, our results were not consistent with the hypotheses existing in the literature. Instead of behaving in a manner that would increase the probability of being picked up by the wind, both mite species reduced their activity by increasing feeding and decreasing walking in the wind presence. This could be interpreted as unwillingness to being dispersed.

Another explanation could be attributed to some of our experimental conditions. Outcomes of this and previous studies on eriophyoid dispersal were drawn from the behaviour of mites held at one particular light, wind speed, temperature and humidity regime. These environmental variables may affect dispersal propensity, distance travelled, locomotor performance or speed^47–50^. In particular, temperature-related factors have a large impact on behaviour of ectotherms since it directly affects metabolic rates, and can alter activity patterns or movement speed^47^. In wind-borne arthropods, dispersal propensity is often strongly correlated with meteorological conditions^51–54^. For example, launch postures of spiders and phytoseiid mites frequently occur in conditions of warm ambient temperature and light breezes^12, 14, 51–55^. Moreover, spider ballooning is known to be positively correlated with increased cloud cover^12^ while wind speed is an important factor determining successful take-off and transfer^52, 54^. The velocity of 4.2 m/s used in our study may seem to be relatively high when compared with studies on other arthropods. For example, spider ballooning was most common at speeds below 3 m/s^52^, and take-off response of phytoseiid mites was maximal at 2 m/s; however their dispersal activity was still very high at speeds of 4 m/s and 8 m/s^55^. Dispersal of other eriophyoid species at wind speeds of 4–4.5 m/s has been shown to be efficient^33, 39, 56^. It is possible that different groups of air-borne arthropods require different wind speed for effective dispersal due to morphological differences between them. Indeed, spiders, phytoseiids, tetranychids and eriophyoids differ in their body size and shape, setae number and length, and use of silk, all of which may influence the probability of being blown away at different wind speeds. Even if they are not phylogenetically related, seeds and spores of some plants and fungi present morphological adaptation to particular wind conditions and their propensity to be blown away tends to decrease at high humidity^57, 58^. Changes in other abiotic factors may also influence the propensity of eriophyid mites to disperse (e.g., mites may be more inclined to engage in wind dispersal when humidity is high and less likely to result in desiccation). It has been suggested that passively dispersed organisms select conditions at take-off that maximize their likelihood of successful dispersal because this will increase their expected fitness after landing^59^. Undoubtedly, understanding the factors that influence the evolution of dispersal propensity and ability is of great interest to evolutionary biologists and ecologists. Thus, further experiments should investigate above mentioned abiotic conditions to appraise the practical relevance of wind tunnel experiments and to test the generality of our findings for these and other species of plant-feeding mites.

However, WCM did respond to the wind presence by increasing the proportion standing erect (Fig. 2), a posture that has been interpreted as a “dispersal position”, but the proportion of standing WCM mites remained low. It is possible that only a small fraction of individuals in a given population of eriophyids are inclined to disperse at any given time. The existence of genetically and phenotypically distinct dispersers and non-dispersers (residents) in a single population has been demonstrated in almost all taxonomic groups, from unicellular organisms to insects and mammals^1, 51, 60^. Dispersers may differ from residents in having physiological, morphological or behavioural traits associated with increased dispersal success, such as fertilization status^61^, tolerance to environmental stress, larger or smaller body size, or greater general activity^1, 62–66^. The small proportion of WCM mites that engaged in the standing-up posture may suggest that in our lab population, there are only a few individuals predisposed to disperse. Also, Cote *et al*.^67^ wondered whether dispersing individuals differ in behaviour from residents across their lives or only when the decision to disperse is about to be made and appropriate dispersal conditions exist. Behaviour of mites in our study was more supportive of the second hypothesis, as they changed their general activity and took up standing-erect position significantly more often when the potential dispersal cue of wind was present.

On the other hand, Melo *et al*.^33^ urged caution in interpretation of standing erect as “the dispersal posture”, since other functions of this behaviour may be possible as well. For example, according to Lindquist & Oldfield^34^, chemosensory sensilla (solenidia) present on the tips of the mite’s legs can better inform the mite about the chemical composition of the environment when the mite stands up and stretches out its legs. Thus, standing erect position may be a helpful behaviour in detecting host plants, predators, competitors or potential mates. The role of this behaviour needs to be studied in more detail in the future.

It has been suggested that the formation of chains is helpful to take-off in aerial dispersal by increasing the overall surface area exposed to wind^24, 68^. In our case, mites did not significantly increase chain formation in the presence of wind. There is a possibility that this behaviour does not facilitate eriophyid dispersal. Similar to the standing-erect position, chain formation may have other functions, e.g. chemical or mechanical communication among individuals (reviewed in Michalska *et al*.)^22^. It is also possible that chain forming occurs only seasonally in wheat-feeding mites (e. g. during crop maturation), as was proposed by Nault & Styer^68^.

Our results showed that both studied eriophyid species (WCM and CRM) expressed similar patterns of behavioural responses, e.g. increased feeding and decreased walking in the presence of wind (Fig. 2). However, there were significant behavioural differences between WCM and CRM (Table 1), reflected mainly by higher general activity of WCM. This result fits with current knowledge of the ecology of WCM MT-1, which is the most important eriophyid agricultural pest as well as the most invasive and globally distributed WCM genotype^69, 70^. High activity of WCM may facilitate its dispersal and spreading as well as its invasive potential. Indeed, it has been shown that many invasive or successful alien species are better dispersers when compared to native or non-invasive ones^71^.

In conclusion, we did not find any strong evidence that two studied species of eriophyids responded to any of the provided cues (wind, fresh plants, potential phoretic hosts) in a way that could be interpreted as intention to disperse, except for the slightly higher proportion of WCM mites standing erect in the presence of wind. This indicates that we need to look more closely at the function of behaviours and activity of these tiny and economically important mites and also raises many interesting questions about how and when they do disperse in field conditions. This is of particular importance in cases of invasive species whose spread may be aided by global changes in environmental conditions.

## Methods

### Experimental plants and stock colonies

In our study we focused on WCM lineage MT-1 (see Skoracka *et al*.)^72^ and CRM *A*. *hystrix* (*sensu stricto*), both of which are well adapted to feed on wheat. All stock colonies as well as all experimental animals in this study were maintained on bread wheat, *Triticum aestivum* L. growing in pots from commercially available seeds. Stock colonies of mites were maintained under laboratory conditions (22–24 °C, photoperiod 16/8, 60% RH) in rearing cages consisting of metal frames wrapped with nylon bags. The stock colony of each species was kept in 7–10 separate pots as a safeguard should one or more fail. To keep the colony homogenous, wheat leaves with mites were transferred between pots every two months. New pots of wheat were put into rearing cages when the previous host plant wilted. WCM and CRM were held in separate laboratories in the Faculty of Biology, Adam Mickiewicz University. WCM MT-1 specimens were collected from wheat in Choryń (loc. 52°02′36″N 16°46′02″E, GenBank Acc. No: JF920077), and specimens of CRM were collected from quack grass, *Elymus repens* (L.) Gould, in Poznań (loc. 52°28′04″N 16°55′36″E, GenBank Acc. No: FJ387550.1). Field-collected specimens were transferred under a stereomicroscope from the collected plant shoots to potted wheat using an eyelash glued to a preparatory needle. In the course of establishing each stock colony several specimens were collected into Eppendorf tubes containing 180 µL of ATL buffer (Qiagen GmbH) for DNA extraction and subsequent DNA sequencing to confirm the molecular identification of studied mites. For DNA isolation a non-destructive method of DNA extraction was performed^73^. The cytochrome c oxidase subunit I (COI) gene fragment was amplified by PCR with the degenerate primers bcdF01 and bcdR04 following Skoracka & Dabert^35^. Subsequently, the PCR amplicon (650 bp) was sequenced with BigDye Terminator v3.1, in accordance with the manufacturer’s instructions, and products from sequencing reactions were analyzed on an ABI Prism 3130XL or 3730 Analyzer (Applied Biosystems). Trace files were checked, edited and aligned using MEGA6 (Tamura *et al*.^74^), and subsequently compared with reference sequences of each studied species downloaded from GenBank. This procedure was repeated every few weeks to test for evidence of contamination of one species’ colony by the other species. No such contamination was ever observed.

*Anaphothrips obscurus* Müller (Thysanoptera), the most abundant thrips species on wheat and other grasses in Poland^75^, was used as a potential dispersal vector for mites. We chose *A*. *obscurus* because it is associated with grasses including cereals and on our preliminary observations of 12 530 field-collected grass shoots: this species was the most common winged insect on wheat infested by WCM and CRM. Moreover during inspections we found WCM and CRM attached to the thrips specimens. Additionally, *A*. *obscurus* inhabits and explores the same plant microhabitats as WCM and CRM (e.g., leaf sheaths and bases, leaf furrows, inner parts of the leaf), which increases the likelihood of contact between eriophyoids and thrips compared to other insects (e.g., bees and syrphids) that visit grasses sporadically and land mostly on the outer leaf surfaces and on flowers. Other authors^40^ have found grass-associated eriophyoid mites on thrips. Thrips were collected from quack grass in Huby Moraskie (52°28′04.3″N 16°55′36.2″E). The plant shoots were inspected using a stereo-microscope (Olympus SZ40) and thrips were transferred into potted wheat using a small paintbrush. For species identification, thrips specimens were mounted on microscopic slides and examined under a compound microscope (Olympus BX41) and identified using Mirab-Balou *et al*.^76^.

### Experimental design

In stock colonies, mites were allowed to multiply for ca. 30 generations under laboratory conditions (22–24 °C, photoperiod 16/8, 60% RH), ensuring a sufficient population size for the experiments (mean number of mites per leaf ~1500, which according to our earlier observations can be considered as highly crowded conditions). We examined the stock colonies in pots every few days and for experiments we chose leaves with similar (ca. 1500 specimens) densities. We ensured that all tested individuals within the species were descendants from the same stock colony in which population density was relatively stable. Therefore, variation in population density was not a factor influencing the behaviour of our experimental specimens. Additionally, leaves taken from the same pot for the purpose of behavioural observations were exposed to different treatments of the experiment, which ensures relatively even distribution of population densities across treatments. Additionally, we found that mites behavioural responses were independent of density (see Supplementary Methods online and Supplementary Figs S1 and S2).

All observations were carried out using a stereo-microscope (Olympus SZ51) and the whole procedure was identical for both eriophyid mite species. A fragment of wheat leaf (1.5 cm length) was cut and glued to a circular piece of adhesive tape (∅ 2.5 cm) to provide an arena for observations. The slow drying of the leaf during the experiment was expected to act as a signal for mites to disperse. Mean density on the cut leaves was 73.1 (95% CI: 61.2–86.5) for CRM and 132.2 (95% CI: 112.9–153.6). Mites could not walk cross the band of adhesive tape surrounding the leaf fragment. For WCM observations, the leaf fragment was cut near the leaf sheath, which is a place occupied by WCM^69^, whereas for CRM observations, the median leaf part was cut, since this species inhabits whole leaves^27^. The tape with the leaf fragment was placed into a small petri dish or on a square glass plate. Just after cutting the leaf, the number of mites of all life stages in the arena was counted to assess the size of the observed group. After 5 minutes, mite behaviour was surveyed by counting the number of individuals engaged in each activity listed below (see *Testing influence of potential dispersal cues* section). During each observation period, the behaviour of each mite was checked once and immediately recorded. Each observation period was replicated at least 20 times for each mite species and for each dispersal cue plus control. Each replicate was observed for three minutes. Observations were carried out between noon and 4 p.m. under room temperature (22–24 °C) and constant artificial light conditions (337.5 ± 4.95 lux during setting up the experiment, 599.0 ± 7.36 lux during observations under stereomicroscope). Lux was measured using Voltcraft BL-10 L Luxmeter.

### The behavioural categories

To describe mite behaviour we used four previously documented activities^22, 23, 26, 27, 56, 77–81^:

- Feeding: mite lies immobile on the leaf surface, feeding on plant tissues;
- Walking: mite moves on the leaf surface;
- Standing erect: mite stands on its anal lobes and moves the legs rapidly, or (much less commonly) mite lifts its hind body off of the leaf surface when standing on the legs (Fig. 1c);
- Chain formation: three or more mites contact each other using their anal lobes, setae or legs. Mites were counted as individuals involved in each chain formation event (Fig. 1d).

Standing erect and chain formation have been explicitly interpreted as facilitating dispersal^23–26^.

### Testing influence of potential dispersal cues

We expected that drying of the leaf fragment during the observation time would be an important background factor encouraging mites to initiate dispersal behaviour, and tested three other potential dispersal cues: (i) wind; (ii) presence of a potential insect vector; iii) presence of fresh plant material. As a control, mites were observed on leaf fragments with none of the other three potential dispersal.

#### Wind

Observations were carried out within a wind tunnel equipped with a fan (design modified from Melo *et al*.)^33^. The tunnel consisted of a transparent PVC (polyvinyl chloride) tube (∅ 4.5 cm, 20 cm length) with a free air inlet at one end and with the other end connected to an axial fan (AC Axial Fan DP202A212, Sunon) via a PVC tube. The fan was connected to a controller (AVT1813, AVT) that allowed for wind-speed regulation. At the distance of 1.5 cm from the free inlet, an open window (∅ 3.0 cm) at the top of the tube was present that allowed stereomicroscope observations. The experimental arena, consisting of the leaf fragment glued to the circle of adhesive tape on a square glass plate (12.25 cm^2^), was put in the tunnel under the open window. Wind speed in the tube was measured using a digital anemometer (Benetech model GM816) placed at the free end of the wind tunnel. The wind blew with a constant speed of 4.2 m/s (=15.12 km/h). This speed was chosen based on our preliminary observations showing that the optimal conditions inside the tunnel (those allowing mites to be dispersed but not forcing them to be blown) was between 3.5 and 4.5 m/s. We also followed previous research indicating that a speed around 4 m/s is suitable for effective eriophyid dispersal^33, 56^. Generally, it was difficult to calibrate whether the air currents inside the tunnel exactly matched that of atmospheric conditions. However, this constraint is present in all experimental set-ups using wind tunnels. Our equipment was built according to similar designs successfully used for studying wind dispersal in mites, including eriophyoids^33, 55^. Our replication of these conditions allows for comparability among studies.

#### Vector

One winged adult thrips (*Anaphothrips obscurus)* was transferred from the stock colony to the leaf fragment with the use of a small paintbrush and needle. During the experimental observations, thrips either walked on the leaf surface or they fed on the plant, occasionally changing feeding position. The thrips specimen was present on the leaf during the whole experiment.

#### Fresh plant material

A new clean wheat leaf cut into small fragments (1.5 cm length) and pressed on to the adhesive tape so that the pieces adhered were put near the leaf fragment with mite population on each side, with no gap between them.

### Data Analysis

To investigate whether a specific behaviour was linked with one or more of the potential dispersal stimuli and whether the response differed between the studied species, a generalized linear model (GLM) was used with quasi-binomial distribution for proportions and the logit link function. The response variable was the proportion of mites performing a given behaviour during an experimental trial, and predictors were treatment (wind, vector, fresh plant material, control), species (CRM, WCM) and treatment x species interactions. For each behavioural category a separate model was built.

For each mite species separately, the contrasts between each treatment and respective control were compared, controlling the error rate by applying the method proposed by Hothorn *et al*.^82^. Statistical analyses were performed with R 3.3.1^83^.

### Data Availability

The datasets generated and analysed during the current study are available in the Zenodo repository [https://zenodo.org/record/437136] under: doi:10.5281/zenodo.437136.

## Electronic supplementary material

Supplementary information
