# Supplementary material for: Behavioural responses to potential dispersal cues in two economically important species of cereal-feeding eriophyid mites
Source: Sci Rep. 2017 Jun 20;7:3890. doi: 10.1038/s41598-017-04372-7 (PMC5478656; doi:10.1038/s41598-017-04372-7)
Supplement: Supplementary file 1 — Supplementary information [file 41598_2017_4372_MOESM1_ESM.pdf]

Behavioural responses to potential dispersal cues in two economically important species of cereal-feeding eriophyid mites

Agnieszka Kiedrowicz*[1], Lechosław Kuczyński[1], Mariusz Lewandowski[2], Heather Proctor[3], Anna Skoracka[1]

[1] Population Ecology Lab, Adam Mickiewicz University in Poznań, Umultowska 89, 61-614 Poznań, Poland

[2] Department of Applied Entomology, Faculty of Horticulture, Biotechnology and Landscape Architecture, Warsaw University of Life Sciences - SGGW, Nowoursynowska 159, 02-776 Warsaw, Poland

[3] Department of Biological Sciences, University of Alberta, Edmonton, Alberta, Canada T6G 2E9

* Corresponding author: kiedra@amu.edu.pl

Supplementary Information

Supplementary Table S1. Mean probabilities (with confidence intervals, CI) of CRM (cereal rust mite, *Abacarus hystrix*) and WCM (wheat curl mite, *Aceria tosichella*) specimens indicating given behavioural response in four treatments (control, new plant, vector and wind).

behavioural response	species	dispersal cue	mean probabilities (%)	CI	
				2.5%	97.5%
feeding	CRM	control	66.50	58.86	73.60
		new plant	71.58	63.43	78.88
		vector	56.03	48.11	63.74
		wind	83.34	76.08	89.22
	WCM	control	13.59	8.61	19.88
		new plant	22.01	16.12	28.77
		vector	20.26	13.49	28.40
		wind	37.23	29.42	45.52
walking	CRM	control	32.91	26.44	39.84
		new plant	26.41	19.91	33.69
		vector	40.79	33.85	47.98
		wind	14.10	9.11	20.33
	WCM	control	83.28	77.19	88.37
		new plant	71.96	65.42	77.94
		vector	73.31	65.38	80.34
		wind	54.96	47.32	62.44
standing erect	CRM	control	1.42	0.91	2.08
		new plant	0.97	0.53	1.60
		vector	1.37	0.87	2.03
		wind	1.78	1.14	2.61
	WCM	control	1.48	0.94	2.19
		new plant	1.10	0.67	1.67
		vector	2.07	1.35	3.02
		wind	4.04	3.09	5.16
chain formation	CRM	control	1.32	0.85	1.94
		new plant	0.97	0.55	1.57
		vector	1.34	0.87	1.97
		wind	1.03	0.58	1.67
	WCM	control	2.43	1.74	3.27
		new plant	0.86	0.50	1.37
		vector	1.97	1.29	2.86
		wind	2.93	2.15	3.86

Supplementary Table S2. Simultaneous tests for general linear hypotheses. Abbreviations: CRM - cereal rust mite, *Abacarus hystrix*; WCM - wheat curl mite, *Aceria tosichella.*

behavioural response	mite species	dispersal cue (compared to control)	estimate	std. error	z value	Pr(>|z|)
feeding	CRM	new plant	0.2379	0.2585	0.920	0.8946
		vector	-0.4436	0.2353	-1.886	0.2698
		wind	0.9244	0.2963	3.120	0.0103
	WCM	new plant	0.5845	0.3097	1.887	0.2690
		vector	0.4796	0.3411	1.406	0.5824
		wind	1.3274	0.3025	4.388	<0.0010
walking	CRM	new plant	-0.3122	0.2394	-1.304	0.6594
		vector	0.3395	0.2161	1.571	0.4657
		wind	-1.0948	0.2835	-3.861	<0.0010
	WCM	new plant	-0.6630	0.2599	-2.551	0.0571
		vector	-0.5954	0.2843	-2.094	0.1762
		wind	-1.4065	0.2585	-5.441	<0.0010
standing erect	CRM	new plant	-0.3828	0.3510	-1.090	0.8051
		vector	-0.0368	0.3034	-0.121	1.0000
		wind	0.2283	0.3010	0.759	0.9546
	WCM	new plant	-0.3046	0.3200	-0.952	0.8817
		vector	0.3416	0.3015	1.133	0.7783
		wind	1.0286	0.2563	4.013	0.0004
chain formation	CRM	new plant	-0.3104	0.3436	-0.903	0.9162
		vector	0.0170	0.2996	0.057	1.0000
		wind	-0.2534	0.3436	-0.737	0.9664
	WCM	new plant	-1.0482	0.3040	-3.448	0.0033
		vector	-0.2129	0.2646	-0.805	0.9496
		wind	0.1922	0.2251	0.854	0.9343

Supplementary Methods

We made tests to check whether the observed behavioural responses were density-dependent. This was based on the assumption that if a given behaviour is modified by population density, there should be a non-linear relationship between the number of individuals showing a particular behaviour vs. the total no. of individuals in the population. If some behaviour is independent of population density, then a constant proportion of individuals will express this behaviour independent of population size, which will result in a straight line describing this relationship (with the slope being just a mean proportion of this category in the whole population). If there is a density-dependent behaviour, this line will be convex (higher densities suppress the behaviour) or concave (higher densities incite the behaviour).

We tested this idea by relating the number of individuals expressing a given behaviour to the total number of observed individuals and fitting a power function to this relationship. Then we tested if the exponent of the power function was statistically different from one. Due to zero inflation, this analysis could be done only for the most frequently observed behavioural category, i.e. "feeding". However, we believe this behaviour is a good reflection of overall inactivity, which obviously is strictly (but negatively) correlated with dispersal propensity. We expected that the more crowded the population, the fewer animals would feed (so, the proportion of feeding should decrease with density resulting in a convex function). We felt that it was less likely that the function would be concave, meaning that the more crowded the population, the more likely it would be for animals to feed (proportion of feeding would increase with density). If the value of exponent equals one, the proportion of feeding is constant and independent of population density. Supplementary figures S3 and S4 below show results of this analysis. We found no significant non-linear relationships, suggesting that behavioural responses were independent of density (at least for the "feeding" category).

Supplementary Figure S1.

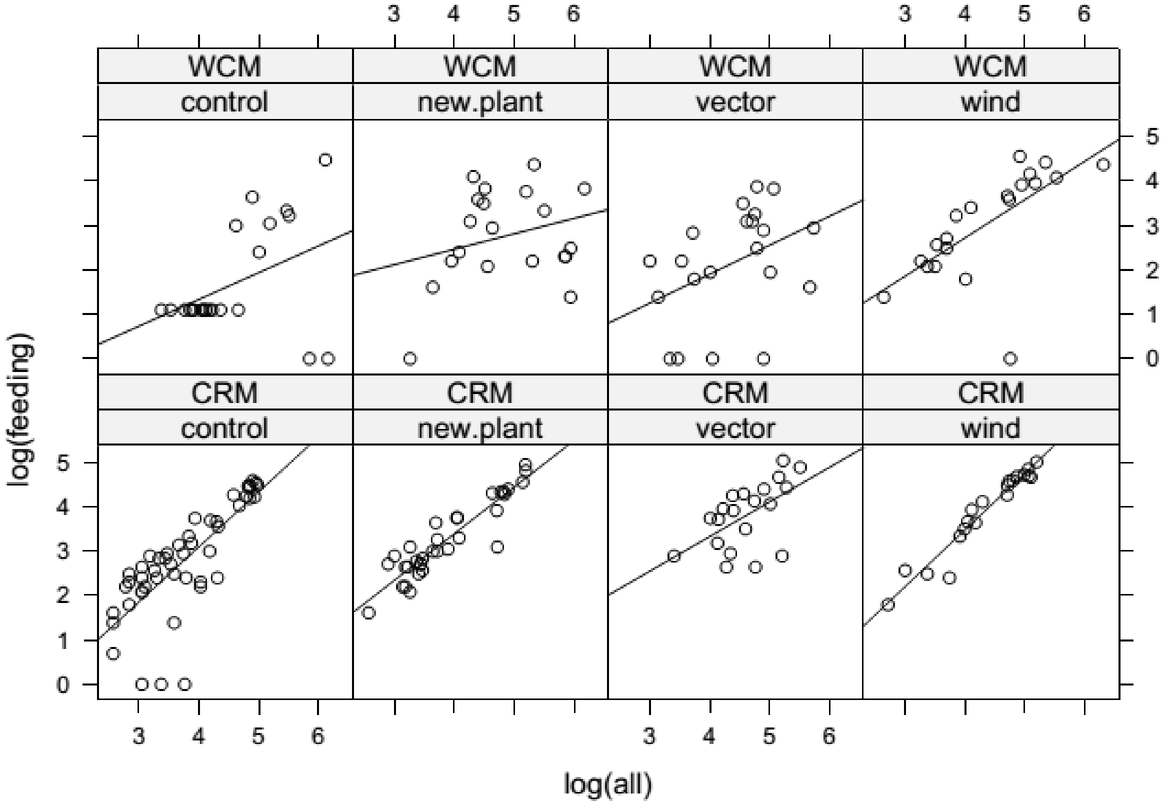

Supplementary Figure S1. Relationships between the total number of observed mites and the number of mites in the "feeding" category for different experimental treatments and for each species separately. Both axes are log-transformed. There is no clear evidence that any of these functions are non-linear, which suggests that the proportion of specimens feeding is constant over the population density range. Abbreviations: CRM - cereal rust mite, *Abacarus hystrix*; WCM - wheat curl mite, *Aceria tosichella.*

Supplementary Figure S2.

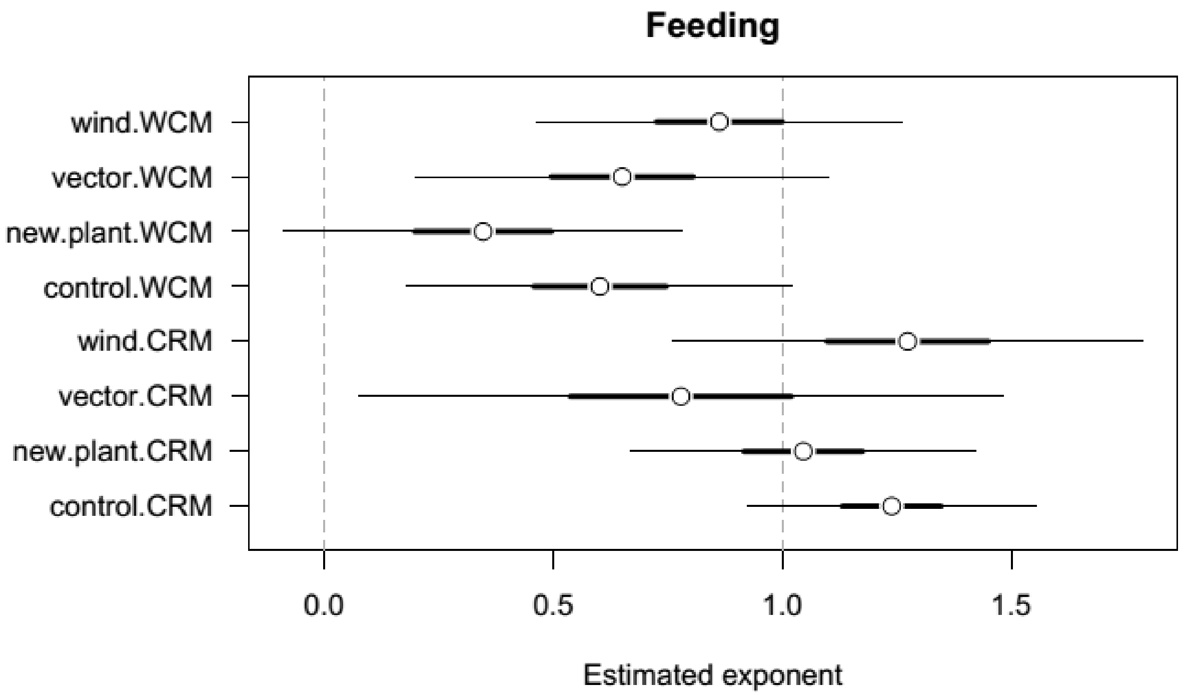

Supplementary Figure S2. Results of testing the null hypothesis that the exponent of a power function equals one. Points denote estimated values, thick lines are 50%, and thin lines are 95% confidence limits for this estimate. For 7 out of 8 combinations of species and treatments, the 95% confidence intervals include 1.0, which means that the null hypothesis cannot be rejected (at the alpha=0.05 level) and that the relationship does not deviate from linearity. The only exception is a new plant treatment in WCM, but the exponent is not different from zero in this case, meaning that this relationship is not significant at all. Abbreviations: CRM - cereal rust mite, *Abacarus hystrix*; WCM - wheat curl mite, *Aceria tosichella.*
